# Interventions to Improve Hydration in Older Adults: A Systematic Review and Meta-Analysis

**DOI:** 10.3390/nu13103640

**Published:** 2021-10-18

**Authors:** Chevonne Bruno, Annaleise Collier, Margaret Holyday, Kelly Lambert

**Affiliations:** 1 School of Medicine, University of Wollongong, Wollongong, NSW 2522, Australia; cb966@uowmail.edu.au; 2 Nutrition and Dietetics Department, Prince of Wales Hospital, Randwick, NSW 2031, Australia; annaleise.collier@health.nsw.gov.au (A.C.); margaret.holyday@health.nsw.gov.au (M.H.); 3 Illawarra Health and Medical Research Institute, Wollongong, NSW 2522, Australia

**Keywords:** dehydration, fluid, beverages, geriatric, inpatient, institutionalized, elderly, systematic review, meta-analysis

## Abstract

Dehydration is common in the elderly, especially when hospitalised. This study investigated the impact of interventions to improve hydration in acutely unwell or institutionalised older adults for hydration and hydration linked events (constipation, falls, urinary tract infections) as well as patient satisfaction. Four databases were searched from inception to 13 May 2020 for studies of interventions to improve hydration. Nineteen studies (978 participants) were included and two studies (165 participants) were meta-analysed. Behavioural interventions were associated with a significant improvement in hydration. Environmental, multifaceted and nutritional interventions had mixed success. Meta-analysis indicated that groups receiving interventions to improve hydration consumed 300.93 mL more fluid per day than those in the usual care groups (95% CI: 289.27 mL, 312.59 mL; I^2^ = 0%, *p* < 0.00001). Overall, there is limited evidence describing interventions to improve hydration in acutely unwell or institutionalised older adults. Behavioural interventions appear promising. High-quality studies using validated rather than subjective methods of assessing hydration are needed to determine effective interventions.

## 1. Introduction

Dehydration is the most common fluid and electrolyte complication amongst the elderly [1]. It is highly prevalent in hospitalised and institutionalised settings [2]. Nursing homes have also identified inadequate fluid intake amongst 50–90% of residents [2]. Similarly, in an Australian geriatric rehabilitation ward, almost one in five patients were found to be dehydrated [3]. Patients with dysphagia are particularly susceptible to the development of dehydration [4,5]. This is often attributed to poor compliance and low satisfaction rates with thickened fluids reported by patients with dysphagia [5] A study in an acute hospital setting demonstrated that patients on thickened fluids consumed only 23.4% of their fluid requirements on average [6]. Furthermore, it has been shown that up to 55% of individuals with dysphagia are at risk of dehydration, which can lead to decreased quality of life and increased healthcare costs [7]. 

Dehydration increases risk of morbidity and mortality [8]. This is because lower hydration levels are associated with incidences of acute confusion, constipation, urinary tract infections (UTIs), exhaustion, falls and delayed wound healing [9,10]. Dehydration has also been correlated to longer hospital stays with the annual cost estimate for a primary diagnosis of dehydration being >$1.14 billion 1999 USD [11,12]. Older adults are at increased risk of dehydration due to age related physiological changes, such as decreased thirst sensation and impaired renal function [11]. This risk is often exacerbated in those with mental illness or stroke [11]. 

The definition of dehydration has been debated as it can be often generalised to describe any fluid imbalance in any fluid compartment [1]. A proposed definition is that dehydration is a complex condition resulting in a reduction of total body water [9]. More expansive definitions can be found when accounting for varying effects in the extracellular compartment (isotonic, hypertonic, hypotonic) [13]. There is also a lack of consensus about which measure should be considered the gold standard for determining hydration status [14,15]. A recent Cochrane systematic review and multidisciplinary consensus statement determined serum osmolality as the gold standard [13,16]. When this method is not readily available it should be substituted with a specific formula (the Khajuria–Krahn formula [17]) to calculate plasma osmolarity [13]. Other techniques for determining dehydration provide singular measures of a complex matrix as opposed to capturing the whole fluid regulation process and some measures may not be appropriate in the elderly population due to declining renal function [15,18]. 

Despite the high prevalence of dehydration, there is limited consensus on the success or efficacy of interventions to improve hydration status. Previous systematic reviews on the topic are more than 15 years out of date [19] or were confined to the long-term care setting [20]. The purpose of this systematic review was therefore (i) to evaluate the impact of interventions to improve hydration in acutely unwell or institutionalised older adults (ii) and to describe the association between interventions to improve hydration and hydration linked events (constipation, falls, UTIs) and patient satisfaction. 

## 2. Materials and Methods

Reporting of this systematic review follows the Preferred Reporting Items for Systematic Reviews and Meta Analyses (PRISMA) guidelines. The protocol for this systematic review was registered with the International Prospective Register of Systematic Reviews (PROSPERO) on 13 August 2020 (registration number: CRD42020197422).

### 2.1. Search Strategy

A systematic search was conducted to identify studies that implemented an intervention to improve hydration or fluid intake in acutely unwell or institutionalised older adults. The search strategy was guided by advice from a librarian and a similar systematic review conducted previously [20]. A preliminary search of the literature was completed to help refine key search terms. The final search terms for use in CINAHL database are shown in Supplementary Material Table S1 and were modified to suit each database. Key search terms involved use of MeSH terms for the population including “Aged” or “Aged or geriatrics”; intervention terms relating to “fluid therapy”, “drink”, “fluid”, and outcome search terms relating to “dehydration”, “hypovolemia” or “hypernatremia”. These search terms were entered into four databases (CINAHL, Medline, Scopus and Web of Science). Hand searching of the reference lists of previous relevant systematic reviews was also conducted to identify additional articles for inclusion. Inclusion and exclusion criteria are summarised in Table 1. 

### 2.2. Eligibility Criteria 

Articles for inclusion in this review included acutely unwell patients (≥65 years) in hospital settings or residents (≥65 years) in an institution such as a nursing home or long-term rehabilitation setting, and papers written in the English language. Intervention studies were eligible for inclusion. Studies were excluded if they involved older adults living in the community, palliative patients, people <65 years, strategies involving parenteral nutrition, enteral nutrition or intravenous fluids or the outcome did not relate to hydration or fluid intake. Case reports, review articles, abstracts, conference proceedings and observational studies were excluded from this review. Articles that included secondary outcomes on hydration linked events (HLEs), such as constipation, falls, urinary tract infections were included. Patient satisfaction with the intervention were noted where a primary outcome was described. 

### 2.3. Data Extraction

The results from the database searches were downloaded into Endnote X9 (Thompson Reuters, New York, NY, USA). Duplicates were removed. Titles and abstracts were screened by two people (CB, KL) to determine eligibility into the systematic review. Information extracted from the articles was conducted by two people (CB, KL) and included: author, country, study design, setting, participant characteristics, intervention, duration, outcome(s) and method of assessment. Interventions were grouped into four categories: behavioural, environmental, multifaceted and nutritional. 

### 2.4. Meta-Analysis

Studies were eligible for meta-analysis if more than two randomised controlled trials on the same outcome were available and (i) reported useable data in a compatible metric (ii) had a matched control group assessed at the same time. RevMan5 (Review Manager (RevMan) [Computer program]. Version 5.4.1, The Cochrane Collaboration, 2020) was used to conduct analyses. Mean difference was used and the standard deviation of the difference was calculated using the formula SD = √SDbaseline^2^ + SDpost^2^ − (2 × r × SDbaseline × SDpost), where r is assumed to be 0.5. To account for heterogeneity between the studies, a random effects model was used. Statistical significance was set at *p* < 0.05. Variance between studies was evaluated and reported as I^2^, which indicates the degree of variance resulting from between study heterogeneity, where a high score closer to 100 indicates high heterogeneity between studies. 

### 2.5. Assessment of Study Quality

The Academy of Nutrition and Dietetics Criteria Checklist for primary research was used to evaluate study quality [14] This tool provides an overall rating of positive, negative or neutral. Two independent reviewers assessed the risk of bias. A positive rating indicates the article has clearly addressed issues of bias, generalisability, inclusion/exclusion criteria, data collection and analysis. A negative rating indicates these issues have not be sufficiently addressed. A neutral rating implies some areas may be unclear and therefore is not classified as a strong or weak study. 

## 3. Results

### 3.1. Study Selection

**Figure 1:**
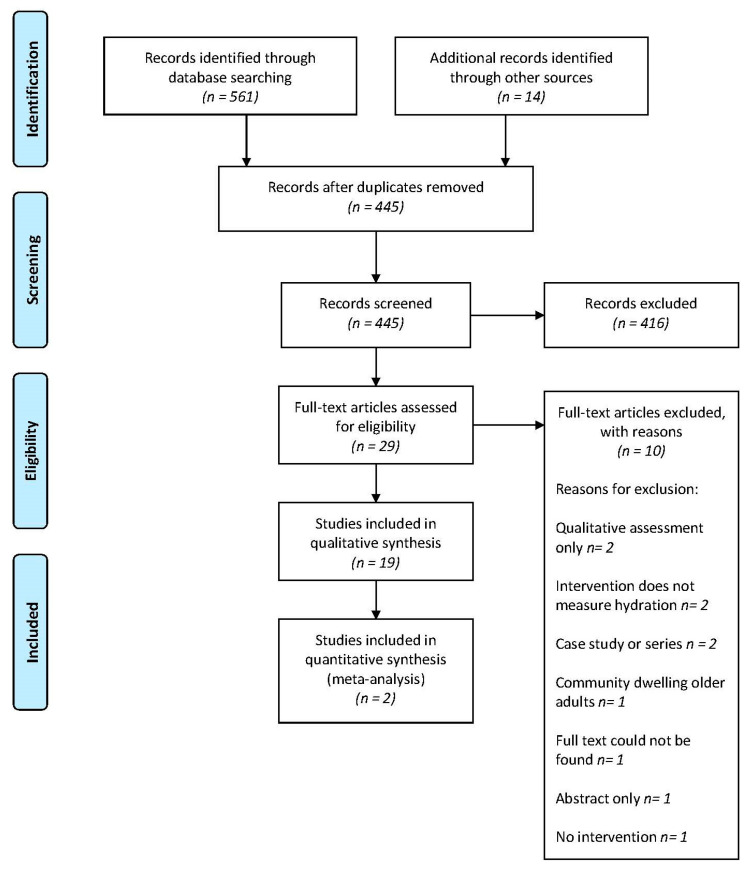
PRISMA Flow chart of study selection.

A total of 575 articles were identified in the database searches as well as through hand searching reference lists (Figure 1). After exclusion of duplicates, 445 articles were screened, and 29 full text articles were reviewed for eligibility. A total of 19 articles [7,21,22,23,24,25,26,27,28,29,30,31,32,33,34,35,36,37,38,39] were included in the review and two articles [23,31] were eligible for a meta-analysis. 

### 3.2. Study Characteristics 

Characteristics of the included studies are shown in Table 2. The studies were grouped into four categories according to the nature of the intervention: behavioural, environmental, multifaceted and nutritional interventions. Seven studies [21,22,26,27,28,29,36] (37%) utilised a pre-test post-test design and five studies [23,24,25,33,39] (26%) were randomised controlled trials. Four studies [30,35,37,38] (21%) were randomised controlled crossover trial, two studies [31,32] were cluster controlled trials and one study [7] was a retrospective analysis. Nine studies [7,22,25,26,27,29,33,35,38] (47.4%) were conducted in the United States of America, four [21,28,32,39] (21%) in the United Kingdom and two [23,24] (%) in Australia. One study was conducted in each of Canada [37], Ireland [30], Japan [36] and Taiwan [31].

### 3.3. Risk of Bias

Assessment of the quality of the included studies are shown in Supplementary Table S2. Evaluation of the risk of bias was rated as neutral for nine studies [21,22,27,28,29,30,32,35,36] (47.3%) and positive for ten studies [7,23,24,25,26,31,33,37,38,39] (52.6%). Of the nine neutral studies, information on selection of study participants, use of blinding and outcome measures were most frequently reported as unclear therefore contributing to the neutral ratings. Three of the studies rated as positive [25,26,31] had minor discrepancies with validity questions relating to selection criteria, comparable groups and intervention. However, these studies were determined to have a low risk of bias overall. 

### 3.4. Participant Characteristics 

A total of 978 participants were reported across the nineteen included studies. The sample size of the included studies ranged from 9 to 122 (average sample size was 54 participants). Thirteen studies [7,22,23,25,26,30,31,32,33,36,37,38,39] (68.4%) had a higher number of females than males. Cognitive impairment was present in twelve studies [23,24,25,26,27,29,31,32,33,35,36,39] (63%) and this varied from mild to severe. Six studies [7,22,28,30,37,38] did not report the cognition status of participants. Fifteen studies [21,22,25,26,28,29,30,31,32,33,35,36,37,38,39] (79%) were undertaken in nursing homes or long-term care facilities. Of the six studies [7,23,24,27,30,39] conducted in hospital settings, four [7,23,24,30] included patients with dysphagia. These patients were from stroke units, rehabilitation facilities and subacute units. 

### 3.5. Hydration Interventions

The results of the interventions can be seen in Table 3. The average intervention duration ranged from 3 days to 12 months. 

### 3.6. Behavioural Strategies 

Seven included studies [21,25,31,33,36,38,39] utilised behavioural interventions. Allen et al. [39] investigated whether participants consuming nutritional supplements through a glass/beaker compared through a straw inserted in the container influenced fluid intake. Residents consumed statistically significantly more supplement drinks from a glass/beaker compared to those who consumed the drink through a straw (64.6 ± 34.3% vs. 57.3 ± 37.0%, *p* = 0.027). 

Bak et al. [21] investigated the design of drinking vessels and their influence on fluid intake. There was a statistically significant increase in fluid intake at breakfast time (*p* = 0.03). However, this result is not clinically significant as the change in intake was only 70 mL in total. 

Lin [31] provided unrestricted drinks choice as part of an intervention to reduce bacteriuria rates in nursing home residents. The change in fluid intake was statistically significant in the intervention group from 1449 mL to 1732 mL (*p* < 0.01). In the control group average fluid intake increased slightly from 1539 mL to 1548 mL however this was not statistically significant (*p* = 0.643). No significance value was determined for the urine specific gravity however the value was slightly lower in the control group than the intervention (1.009 vs. 1.012, respectively). These results fall within the normal range and indicate normal urine osmolality. 

Schnelle et al. [33] offered beverage choices to residents’ multiple times a day to improve fluid intake and compared the results to usual care. The intervention group consumed significantly higher amounts of fluid compared to the control group (399 ± 186 mL vs. 56.2 ± 118 mL, *p* < 0.001). 

Simmons et al. [25] provided daily verbal prompting to drink with the aim to increase fluid intake. Serum osmolality significantly declined in both groups overtime (*p* < 0.05) however changes in BUN:Cr were not significant (*p* > 0.05). There was a significant increase in fluid intake between meals with each phase of prompting (*p* < 0.001). Changes in serum osmolality were small although changes in overall fluid intake were clinically significant across the three phases.

Spangler et al. [38] employed a combined strategy of offering beverage choices and assistance with toileting to nursing home residents every 1.5 h. Urinometer scores at baseline indicated 25% of residents were dehydrated (score > 20) and post intervention all residents had scores < 20 indicating absence of dehydration (*p* < 0.002). 

Tanaka et al. [36] provided residents with beverage choices in between meals and staff offered encouragement to drink with the aim for residents to consume 1500 mL per day. Fluid intake significantly increased after the intervention was implemented (1146.4 ± 365.2) compared to baseline (881.1 ± 263.8, *p* < 0.001).

### 3.7. Environmental Strategies 

Environmental approaches were applied in four studies [22,29,32,35]. Dunne et al. [29] assessed the effect of low and high contrast tableware compared to white tableware on fluid intake in nursing home residents with Alzheimer’s disease. This occurred as two separate studies one year apart. The first study using high contrast red tableware demonstrated a significant mean percent increase of 84% for liquid between baseline and intervention (*p* = 0.001). In the follow up study, the mean percent increase in liquid intake for high contrast blue was 29.8% (*p* < 0.05). 

Holzapfel et al. [35] assigned nursing home residents to three groups where a feeding assistant would provide food and beverages to residents in a specific position (standing, sitting or position chosen by feeding assistant). Statistically significant results were observed with fluid intake at day 5 comparing the control group (choice of position by assistant) and experimental groups (sitting or standing) however all other results at different time points were not statistically significant (*p* > 0.05). 

Kenkmann et al. [32] implemented a program to increase the availability and choice of drinks as well as improve the social and physical environment at mealtimes. Rates of dehydration dropped in both intervention and control care homes (16% to 9% and 46% to 39% respectively) but the significance of this result was not reported. The relative risk of being dehydrated in an intervention home compared to a control home was 0.36 (CI 0.06 to 2.04, *p* = 0.25). There was also a reduced rate of falls by 24% but this was not statistically significant (*p* = 0.06). 

Robinson and Rosher [22] implemented a five week hydration program (increased availability and choice of drinks using a colourful beverage cart) in a nursing home aiming to reach an additional 450 mL of fluid intake at mid-morning and mid-afternoon. The percent of residents meeting the fluid goal was 53% with 24% not meeting the goal every time. No significance value was reported. There was a significant increase in total body water during the program and significant decrease in total body water once the program ceased (*p* = 0.001). The number of bowel movements increased significantly (*p* = 0.04) and the number falls declined significantly (*p* = 0.05). 

### 3.8. Multifaceted Strategies 

Three studies [26,27,28] applied multifaceted interventions to address hydration and fluid intake. Mentes and Culp [26] provided 180 mL of fluid with medication administration, providing drinks in between meals as well as offering a one hour time period where non-alcoholic cocktails are served (also known as happy hour) twice a week in the afternoon. The percent meeting fluid goals, urine colour and specific gravity did not increase significantly for either intervention or control group (*p* = 0.08). Incidence of HLEs was 3 events per 63 days of follow-up for the intervention group and 6 events per 60 days of follow-up for the control group but this was not statistically significant (*p* = 0.39). 

Smith et al. [27] utilised a three-pronged approach (providing flavoured water, using larger cups and increased prompting to drink by nurses) to improve fluid intake. Fluid intake increased with the mean fluid intake at baseline being 1551 mL compared to 2225 mL post intervention. 

Wilson et al. [28] implemented an intervention that included drinks being provided in between main meals, implementation of protected drinks time and increasing choice through a drinks menu. Mean fluid intake at Home A < 1500 mL per day whilst mean fluid intake at Home B was >1500 mL. No statistically significant value was reported. There was no change in the incidence of HLEs however there was a significant decrease in the use of laxatives in both homes (*p* < 0.05). 

### 3.9. Nutritional Strategies 

Five studies [7,23,24,30,37] used strategies targeted at improving overall nutrition and fluid intake in people with dysphagia. Howard et al. [7] conducted a retrospective analysis on an observational study of twenty patients with dysphagia who had received nectar thick and textured thin fluids during their hospital stay. Creatinine and sodium levels significantly increased whilst on the nectar thick diet (*p* = 0.047, *p* = 0.014 respectively). Although serum urea increased when on a nectar thick diet this change was not statistically significant (*p* = 0.07). When patients changed over to the textured thin liquids, serum urea dropped significantly (*p* = 0.06). Creatinine decreased into the normal range, but the change was not significant (*p* = 0.63). 

Karagiannis et al. [23] implemented a water protocol in patients with dysphagia for five days whilst the control group could only consume thickened fluids. Patients with dysphagia had access to both thickened fluids and water between meals. Fluid intake increased significantly in the intervention group receiving the water protocol (1428 ± 7.0 mL to 1767 ± 10.7 mL, *p* < 0.01). The number of lung complications was significantly higher in the intervention group with 6 cases reported compared to zero in the control group (*p* < 0.05). 

McCormick et al. [30] utilised a cross over design to determine if commercially thickened fluids or fluids thickened at the bedside increased fluid intake and influenced rates of constipation. The difference in fluid intake between the two interventions were minimal with 795 mL of pre thickened liquids consumed compared to 785 mL consumed pre thickened drinks at the bedside (*p* = 0.47). No changes in constipation rates were observed. 

Murray et al. [24] applied the same water protocol as previously described by Karagiannis et al. [23] to patients with dysphagia for two weeks. The intervention group had a similar intake to the control group (1103 ± 215 mL, 1103 ± 247 mL respectively, *p* = 0.998). Although, the type of fluid in the intervention was water, it did not lead to an increase in hydration using the BUN:Cr as a proxy for hydration. The control group had a significantly higher incidence of UTIs compared to the intervention group (*p* = 0.024). There were no cases of pneumonia diagnosed during the intervention and no significant differences in constipation were discovered between both groups (*p* = 0.733). Taylor and Barr [37] implemented a crossover study to assess if a 3 day meal pattern compared to a five day meal pattern improved fluid intake. Fluid intake was higher at with five meals (698 ± 156 mL) compared to three meals (612 ± 176 mL, *p* = 0.003). 

### 3.10. Hydration Linked Events

Eight studies [22,23,24,26,28,30,32,33] used HLEs as an indirect measure of hydration status and intervention effectiveness. HLEs measured included lung complications, falls, constipation, UTIs, laxative and antibiotic use. Five studies [22,24,26,32,33] reported improvements in HLEs with implementing a hydration intervention. Two studies [28,30] observed no differences in HLEs, and one study [23] observed adverse effects on lung function from use of water in people with dysphagia. 

### 3.11. Patient Satisfaction

Six studies [7,21,22,23,24,32] gathered information on patient satisfaction. Information was collected in the form of a Likert scale, survey or recording of comments made during the intervention period. Only four studies [7,23,24,32] analysed the satisfaction data and three of the studies [7,24,32] reported no significant differences in satisfaction. One study [23] reported a significant increase in satisfaction with drinks but not in overall positive feeling. 

### 3.12. Meta-Analysis 

**Figure 2:**
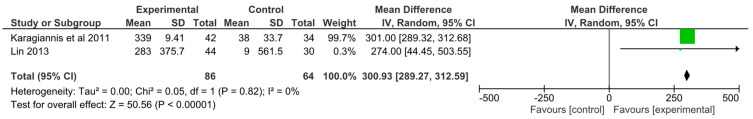
Forest plot of the results from random effects meta-analysis on fluid intake. Results are presented as mean difference (MD) between baseline and post intervention with the corresponding 95% confidence interval.

Only two studies were able to be included in the meta-analysis. Karagiannis et al. [23] implemented a nutritional intervention and Lin [31] implemented a behavioural intervention. Overall, groups receiving interventions to improve hydration consumed 300.93 mL more fluid per day than those in the intervention groups (95% confidence interval 289.27 mL, 312.59 mL, I^2^ = 0%, *p* < 0.00001). The forest plot for this analysis is shown in Figure 2.

## 4. Discussion

This systematic review investigated the impact of interventions on improving hydration in older adults in nursing homes and hospital settings. Interestingly, only nineteen studies were eligible to be included in this review, which is concerning as dehydration is known to be a key problem in the geriatric population [1,40]. The findings of this systematic review are threefold. Firstly, behavioural interventions were associated with positive effects on hydration and fluid intake whilst environmental, multifaceted and nutritional interventions reported mixed results. Behavioural interventions involving verbal prompting or increased choice and availability of drinks were also associated with improvement in hydration. While metanalyses of outcomes were limited to daily fluid intake due to heterogeneous reporting of outcomes, it was clear that an improvement in fluid intake of 300 mL per day is both clinically as well as statistically significant. HLEs were reported to improve in half of the studies that measured this outcome and satisfaction rates generally observed no significant changes with implementation of an intervention. 

Multifaceted interventions appear to be more difficult to implement than single component interventions as they attempt to address multiple barriers at different levels. In theory, these interventions should be more effective as they target several barriers simultaneously [41]. However multifaceted interventions generally require more resources and are more difficult to sustain [41]. This is consistent with the findings of other interventions implemented in aged care and hospital settings [42,43,44], where resource intensive interventions and organisational support contribute significantly to intervention success [42,43,44]. Interestingly, a previous systematic review on hydration interventions in institutionalised settings found multicomponent interventions showing a trend towards increased fluid intake [20]. This review included non-English articles and the sample was specific to institutionalised settings which may explain the difference in results. 

The second key finding was that few studies used objective measures to measure hydration or used clinical measures for assessment of hydration that are appropriate for the elderly population. Furthermore, only one study used the gold standard of serum osmolality. Aside from serum osmolality and fluid balance charts, no other methods utilised have been validated to measure hydration in the elderly population. Fluid balance charts are considered the best approach for monitoring daily intake but there are obvious concerns around their accuracy as intakes are usually estimated and not precisely measured [45]. In this study, clinical measures were found to be ineffective when compared to the reference standard in older adults [46]. The precision of BUN:Cr ratio and urinary indices is impacted by renal dysfunction which is common in older adults thus is likely to be inappropriate for widespread use [18] Hydration linked events can also be caused by other factors such as medications or health conditions [47]. These concerns surrounding the measurement of hydration are noted in another systematic review investigating hydration in patients with dysphagia due to stroke [48]. The rationale for using these assessment methods was commonly cited as ease of use, or to replicate the method from previous studies or population groups or the methods was validated against another measure [7,22,24,26,28,30,32]. The heterogeneity in clinical assessment methods to evaluate hydration can therefore potentially explain part of the variation in intervention success.

The third key finding of this study is that there is a limited number of studies exploring the topic of hydration in acutely unwell hospitalised patients. Of the five articles conducted in hospital settings, only one study included patients from an acute hospital setting. Patients in acute hospitals typically have a shorter length of stay which can impact the true effect of the intervention. Other common barriers reported in the literature for acute hospital interventions include staff workload, time restraints and staff attitudes towards the intervention [49]. Additionally, a recent qualitative study in an acute hospital indicated that patients felt drinking was a task rather than a pleasurable activity [45]. It was also emphasised that the social interaction that plays a role in drinking was largely underplayed [45]. This point is supported by an unpublished study from a metropolitan teaching hospital in Sydney indicated that a non-alcoholic happy hour trolley that included social interaction was effective at improving fluid intake in older adults.

There are several strengths to this review. A systematic approach to searching databases and the use of multiple databases increased the ability to gather all relevant articles. A clear inclusion criterion was used to determine study eligibility and no study design limiters were applied. This review attempted to capture the evidence from a broad perspective by not focusing on a specific subset of the elderly population. Limitations of this review include restricting the studies to papers written in the English language only. The low-quality rating of studies also suggests the certainty of our findings should be used with caution. The search terms utilised in this review may also not capture all the evidence on hydration interventions in elderly patients or residents. The generalisability of the findings may be impacted by the greater number of the studies conducted in nursing home studies than hospital studies and most hospital studies were conducted in patients with dysphagia due to stroke. 

Several recommendations arise from this research. There is a critical need for more intervention studies using validated methods for assessment of hydration in older adults to determine successful hydration strategies. This would enable comparisons between studies to be made more easily. In addition, studies exploring interventions in the acute hospital population are also required as there were no studies identified in this review that included the general population. Ideally, fluid balance charts and serum osmolality or the use of the Khajuria–Krahn formula [17] should be used to determine intervention success. These methods are considered the best approach when monitoring intake and hydration and can be easily incorporated in routine practice [50]. Studies in this review are charted below using the Behaviour Change Wheel [51] to determine what elements of behaviour change have not yet been targeted (Table 4). There are a lack of interventions addressing education, incentivisation, coercion, training, modelling and restriction aspects of the behaviour change wheel. When planning future interventions these areas should be considered to determine the impact on intervention success. Additionally, the collection of qualitative data with recipients of interventions as well as nursing staff may be beneficial to better understand appropriate methods, perceived barriers and ease of implementation [45]. 

This review examined the impact of interventions to improve hydration in acutely unwell and institutionalised older adults. The major finding was that behavioural interventions utilising verbal prompting and increased availability or choice of drinks were associated with improvements in fluid intake and hydration. When pooled, interventions can improve fluid intake by approximately 300 mL per day. However, further high-quality studies are needed and in additional patient groups and acute care settings. There were limited included studies in this review, of suboptimal quality and large variations in intervention design and evaluation. This highlights the need for more rigorous intervention implementation using validated and population appropriate methods to determine intervention effectiveness. High quality studies using serum osmolality or Khajuria–Krahn [17] formula which can calculate plasma osmolarity in conjunction with fluid balance charts will be of benefit to researchers and clinicians. This is particularly important in the acute clinical setting where a successful intervention could be implemented into practice and result in reduced dehydration related outcomes and length of stay. 

## Figures and Tables

**Table 1:** Inclusion and exclusion criteria.

PICO Component	Inclusion Criteria	Exclusion Criteria
Population	Acutely unwell patients in hospital or residents in nursing homes (>65 years)	Participants below 65 years, palliative patients and older adults living in the community
Intervention	Oral methods to improve hydration or fluid intake	Interventions using parenteral, enteral or intravenous methods
Comparator	Comparator such as usual care	
Outcome	Quantitative measures of hydration status or fluid intake in older adult patients or residents	Any measures not related to hydration status or fluid intake or qualitative assessment only

**Table 2:** Characteristics of included studies (*n* = 19).

Author, Country and Study Type	Setting	Participant Characteristics	Intervention	Duration	Outcome(s)	Description of Outcome Assessment
Behavioural Strategies						
Allen et al. 2013 [39] UK RCT	Eight nursing homes and three hospitals	45 older adults Straw group: 19 (16 females, 3 males) Mean age: 85.4 ± 8.6 years MMSE: 13.1 ± 7.9/30 Glass/Beaker group: 26 (19 females, 7 males) Mean age: 88.4 ± 5.4 years MMSE: 14.6 ± 8.3/30	Group 1: ONS drink with straw inserted Group 2: ONS drink served in a glass or beaker	3x daily for 7 days	Fluid intake measured as the proportion of ONS consumed per day	Amount of ONS consumed was calculated by weighing the supplement and subtracting it from full amount.
Bak et al. 2018 [21] UK Pre-Post study	One nursing home	Phase 1: 37 residents Phase 2: 24 residents Gender: not reported. Age: not reported Residents did not have severe cognitive impairment.	Phase 1: Evaluation of drinking equipment Drinking vessels with different designs selected for evaluation Phase 2: Introduction of new drinking vessels Standard vessels were replaced with vessels rated highest from phase 1. Observations at breakfast on 3 consecutive days.	Phase 1: not recorded Phase 2: 3 days	Fluid intake per day Resident satisfaction	A questionnaire with a five-point Likert scale was used to evaluate ease of vessel use. Baseline fluid intake data compared to fluid intake data with new vessels. Method of collecting fluid intake not recorded. Resident opinions were sought via face to face questioning during and after intervention.
Lin 2013 [31] Taiwan Non randomised clinical trial	Six nursing homes	74 incontinent residents Average age: 75.2 years Intervention: 44 (30 females, 14 males) Control: 30 (15 females, 15 males) SPMSQ: 5.9 ± 3.5	Intervention: Advice to increase daily fluid intake >1500 mL, unrestricted drinks choice Control: Unrestricted drinks, residents could choose type and amount.	6 weeks	Fluid intake per day Hydration status measured by change in urine specific gravity	Intake/output chart was recorded by nursing staff. Method of fluid measurement not recorded. Urine specimens collected at baseline and post intervention. Specimens sent to lab for testing within 2 h of collection.
Simmons, Alessi and Schnelle 2001 [25] USA RCT	Two community nursing homes	63 incontinent nursing home residents. Intervention: 88.7 ± 7.1 years (44 females, 4 males), MMSE = 12.1 ± 7.9 Control: 86.3 ± 6.1 years, (10 females, 5 males), MMSE = 13.9 ± 6.5	Phase 1: four verbal prompts to drink per day Phase 2: eight verbal prompts to drink per day Phase 3: eight verbal prompts to drink per day plus compliance with resident beverage preferences.	32 weeks Phase 1: 16 weeks Phase 2: 8 weeks Phase 3: 8 weeks	Fluid intake per day Hydration status measured by change in BUN:Cr ratio and serum osmolality	Fluid intake between meals measured by research staff using measured drinking cups. Hydration status assessed by BUN:Cr ratio and serum osmolality at baseline, 8 and 32 weeks.
Schnelle et al. 2010 [33] USA RCT	Six nursing homes	112 residents with urinary and faecal incontinence Intervention: 58 (49 females, 9 males) Mean age: 85.8 ± 9.4 Mean MMSE: 12.9 ± 8.4 Control: 54 (44 females, 10 males) Mean age: 86.1 ± 10.5 Mean MMSE: 9.6 ± 8.4	Intervention: to increase fluid intake. Staff offered additional food and fluids between meals Control: ‘usual care’ (not described)	12 weeks (weekdays between 7am-330pm h)	Change in between meal fluid intake daily	Fluid intake was observed at baseline and for 6 meal and 6 in between meal observations. Fluid intake was assessed using a photographic assessment method.
Spangler et al. 1984 [38] USA RCT	One nursing home	16 non ambulatory residents with incontinence Gender: 2 males, 14 females Age: 59–96 years (mean age not reported) Cognition: not reported	Staff offered beverage choice by circulating a beverage cart every 1.5 h. Staff would offer assistance with consuming beverages and with toileting.	50 days	Hydration status measured by change in urine specific gravity	Two urine samples were collected in the morning per resident on the first and third day of data collection. Urine specific gravity was measured with a urinometer.
Tanaka et al. 2009 [36] Japan Pre-Post study	17 nursing homes	122 residents Gender: 18 males, 104 females Mean age: 85.2 years Dementia levels, n: I (mild): 2, II: 18, III: 59, IV (severe): 42	Staff aimed to increase fluid intake to 1500 mL/day by providing beverages at 1000, 1500 and before bedtime. Staff provided encouragement to drink and offered choice.	12 weeks	Mean change in daily fluid intake	3 day baseline intake and 12 week intake assessment completed. Method not recorded.
Environmental Strategies						
Dunne et al. 2004 [29] USA Pre-Post study	One care home	9 males with advanced Alzheimer’s Disease Study 1: mean age 82.7 years Study 2: mean age 83.1 years	Study 1: white tableware (control), high contrast red tableware Study 2 (1 year later): white (control), high contrast blue, low contrast red, low contrast blue.	Study 1: 30 days Study 2: 70 days	Change in mean % of daily fluid intake	Food and fluid intake recorded daily for each participant at lunch and supper. Amount consumed expressed as a percentage of amount served. Amount served weighed in ounces.
Holzapfel et al. 1996 [35] USA RCT	One nursing home	39 residents requiring complete feeding assistance Gender: 3 females, 36 males Mean age: 75 years Cognition: n = 22 had dementia	Intervention: Feeding assistants sat for two weeks, then stood for 2 weeks and crossed over. Control: feeders determined which position to assume at each mealtime	4 weeks (lunch meals Monday–Friday)	Change in daily mean fluid intake at day 1, 5, 10, 15 and 20.	Fluid consumed at lunch meals was recorded by feeder using a likert scale of percentage groups (0–25%, 26–50%, 51–75%, 76–100%)
Kenkmann et al. 2010 [32] UK Non randomised clinical trial	Six care homes	120 residents 85 females, 35 males Mean age: 87 years Two homes were for dementia care	Intervention: restaurant atmosphere, extended mealtimes, increased choice of foods, social experience, encouragement to eat, availability of drinks and snacks Control: ‘usual care’ (not described)	12 months	Number of residents with dehydration Number of falls. -Resident satisfaction	Assessment of dehydration (visual assessment of tongue) by a trained nurse. Number of participants dehydrated at second interview were used to calculate a relative risk of dehydration. Number of falls was collected from care notes. Satisfaction questionnaires were distributed before and after intervention.
Robinson and Rosher 2002 [22] USA Pre-Post study	One nursing home	51 residents 43 females, 8 males Mean age: 83.5 years Cognition: not reported.	Goal: to drink 8 ounces of fluid twice a day. Hydration assistant utilised for fluid administration. Increased choice through using a colourful beverage cart, jugs and glasses.	Baseline: 2 weeks Intervention: 5 weeks Follow-up: 2 weeks	Number of participants drinking extra fluid. Changes in TBW. Number of HLEs (delirium, respiratory infections, constipation, UTIs, falls) Resident satisfaction	Fluid intake recorded at mid-morning and afternoon only. BIA weekly measurements to determine changes in TBW. Number of hydration-linked events tracked through medication and bowel charts. A record of comments made by residents or their family were kept to reflect the value of the program.
Multifaceted Strategies						
Mentes and Culp 2003 [26] USA Pre-Post study	Four long term care facilities	49 residents 25 females, 22 males Intervention: mean age 80.6 years, MMSE = 22 Control: mean age 83 years, MMSE = 24.6	Intervention: Calculation of weight-based fluid intake goal. Providing standardised 180 mL water with medications, fluid rounds twice daily and happy hours or tea time twice a week in the afternoon. Control: Usual care (not described)	8 weeks	Number of HLEs (acute confusion, UTI, respiratory infection) Urine colour and specific gravity % meeting daily fluid goal.	Urine colour determined by standard urine colour chart. Urine specific gravity was determined using Chemstrip Mini Urine Analyzer. Fluid intake records taken at baseline and during intervention (method not described). HLEs were documented when they occurred. Acute confusion assessment was used if acute confusion was suspected.
Smith et al. 2019 [27] USA Pre-Post study	One hospital (geriatric psychiatry unit)	50 patients ≥65 years with a neurocognitive disorder Gender: not reported.	Offered flavoured water, increased cup size and nursing staff to encourage fluid intake.	Baseline: 7 days Follow-up: 7 days	Change in mean daily fluid intake.	Fluid intake form was developed to track daily intake. Standardised cups were used to determine amount of fluid consumed.
Wilson et al. 2019 [28] UK Pre-Post study	Two care homes	Number of residents: not reported Gender: not reported Age: ≥65 years Cognition: not reported	Drinks provided before breakfast and after main meals. Implementation of protected drinks time (PDT). Increasing choice through a drinks menu.	Home A: Drinks before breakfast = 4 days, PDT and drinks menu = 8 weeks. Home B: PDT and drinks menu = 9 weeks	Change in mean daily fluid intake Number of HLEs (UTIs, respiratory infection, falls) Change in laxative and antibiotic use	Fluid intake was measured every 4 weeks by observing volume consumed of 6 randomly selected residents. Information on adverse health events was collected weekly (method not described). Antibiotics and laxatives used were gathered from prescription charts every 4 weeks.
Nutritional Strategies						
Howard et al. 2018 [7] USA Retrospective analysis	One hospital (inpatient rehabilitation facility)	20 patients with dysphagia. 11 females, 9 males Mean age = 79 years Cognition: not reported.	Retrospective analysis of patients who received both nectar thick and textured thin liquids during their hospital stay.	Nectar thick: 8.3 days Textured thin: 5.8 days	Prevalence of dehydration Patient satisfaction	Lab values (Na, serum urea and Cr) were used to determine dehydration. Two clinician initiated questions were used to assess satisfaction.
Karagiannis et al. 2011 [23] Australia RCT	One hospital (subacute unit)	91 patients with dysphagia 34 males, 42 females Intervention: mean age 80 years Control: mean age 79 years 18 participants had Alzheimer’s disease or dementia.	Intervention: consumed thickened fluids but also received water upon request for five days Control: consumed only thickened fluids	Baseline: 3 days Intervention: 5 days	Incidence of lung problems. Change in mean fluid intake Patient satisfaction	Chest status was examined by physicians and core body temperature taken 3 times per day. Daily fluid intake for each participant was recorded. Method of measurement not recorded. Quality of life surveys were administered in the pre and post intervention period.
McCormick et al. 2006 [30] Ireland RCT	One geriatric care facility	11 patients with dysphagia 8 females, 3 males Mean age: 76 years Cognition: not reported.	Week 1–6: Group A received commercially prepared pre-thickened fluids. Group B received drinks thickened at the bedside. Week 6–12: Group B received commercially prepared pre-thickened fluids. Group A received drinks thickened at the bedside.	12 weeks (6 weeks per group)	Difference in amount of thickened fluids consumed. Rates of constipation	Daily assessment of total fluid intake using graduated cups. Constipation rates were recorded using the British stool chart.
Murray et al. 2016 [24] Australia RCT	Two acute hospitals and three rehabilitation facilities	14 patients post stroke with dysphagia 10 males, 4 females Mean age: 80 years 5 patients were cognitively impaired	Intervention: had access to thickened fluids but could also have water between meals. Control: consumed thickened fluids only	2 weeks	Change in mean daily beverage intake Change in hydration Incidence of pneumonia, constipation, UTIs Patient satisfaction	Daily intake recorded on fluid balance charts. Hydration status was assessed using the BUN:Cr ratio. Incidence of UTIs, constipation and pneumonia were recorded. A five question Likert scale survey was completed at weekly intervals throughout the study.
Taylor and Barr 2006 [37] Canada RCT	One extended care facility	31 residents with dysphagia Gender: 5 males, 26 females Mean age: 85 ± 6.4 years Cognition: not reported	Group 1: 5 meals/d for 4 d, Group 2: 3 meals/d for 4 d and then crossover 4 weeks later	4 weeks	Change in fluid intake at mealtimes	Weight of fluid consumed recorded by registered dietitian before and after meals.

Legend: SPMSQ = short portable mental status questionnaire, MMSE = mini mental state examination, RCT: randomised controlled trial; BUN:Cr = blood urea nitrogen and creatinine ratio, UTIs = urinary tract infections.

**Table 3:** Results of included studies (*n* = 19).

	Description of Results
Allen et al. 2013 [39]	Plasma osmolality (mOsm/kg); Lab values; BIA (Ohms); Urinary indices; Number of HLEs: not reported Fluid intake (mL): Glass/beaker consumption: 64.6 ± 34.3% supplement volume; Straw: 57.3 ± 37.0% supplement volume (*p* = 0.027)
Bak et al. 2018 [21]	Plasma osmolality (mOsm/kg); Lab values; BIA (Ohms); Urinary indices; Number of HLEs: not reported Fluid intake (mL): Mean intake at breakfast increased from 139mL (±84 mL) to 205 mL (±12 mL), *p* = 0.003 Patient satisfaction: 20 residents provided feedback; 80% reported they preferred the test mugs to the standard cups. No *p* value reported.
Dunne et al. 2004 [29]	Plasma osmolality (mOsm/kg); Lab values; BIA (Ohms); Urinary indices; Number of HLEs: not reported Study 1: Mean 84% fluid increase per day between baseline and intervention (*p* = 0.001). Study 2: Mean 29.8% fluid increase per day for high contrast blue (*p* < 0.05). Low contrast blue and red interventions were ineffectual. No volume (mls) reported.
Holzapfel et al. 1996 [35]	Plasma osmolality (mOsm/kg); Lab values; BIA (Ohms); Urinary indices; Number of HLEs: not reported Group 1 = control, Group 2 = stand then sit, Group 3 = sit then stand Group 1 and 2, Group 1 and 3, Group 2 and 3, respectively Day 1: *p* = 0.600, *p* = 0.209, *p* = 0.533 Day 5: *p* = 0.019 *, *p* = 0.012 *, *p* = 0.776 Day 10: *p* = 0.597, *p* = 0.625, *p* = 0.743 Day 15: *p* = 0.506, *p* = 0.830, *p* = 0.625 Day 20: *p* = 0.707, *p* = 0.972, *p* = 0.710 * statistically significant result *p* < 0.05
Howard et al. 2018 [7]	Plasma osmolality (mOsm/kg); BIA (Ohms); Fluid intake (mL); Urinary indices; Number of HLEs: not reported Lab values: Nectar thick diet—Serum urea rose from 8.2 mmol/L to 9.6 mmol/L (*p* = 0.07). Cr rose from 104.3 umol/L to 153.8 umol/L (*p* = 0.047) Na levels peaked after a nectar thick diet (*p* = 0.014) Nectar thick to textured thin liquids—Serum urea dropped into normal range (*p* = 0.006). Cr decreased into normal range but was not significant (*p* > 0.05). Patient satisfaction: Patients reported being able to consume a greater variety of liquids (*p* = 0.06). They also reported that their thirst was quenched better when receiving textured thin liquids compared to nectar thick fluids (*p* = 0.0059)
Karagiannis et al. 2011 [23]	Plasma osmolality (mOsm/kg); Lab values; BIA (Ohms); Urinary indices: not reported Fluid intake (mL) preintervention period: *Intervention:* 1428 ± 7.0 mL per day; *Control:* 1340 ± 9.5 mL per day Fluid intake (mL) Intervention period: *Intervention:* 1767 ± 10.7 mL per day (*p* < 0.01); *Control:* 1378 ± 33.7 mL per day HLEs (Incidence of lung complications): *Intervention:* 6 patients (14.3%); *Control:* 0 patients (*p* < 0.05) Patient satisfaction: The intervention group reported higher levels of satisfaction than the intervention group (*p* < 0.001). General positive feeling was higher than control group but was less than in pre intervention period (*p* = 0.111)
Kenkmann et al. 2010 [32]	Plasma osmolality (mOsm/kg); Lab values; BIA (Ohms); fluid intake (ml), Urinary indices: not reported HLEs: intervention group had reduced rate of falls by 24% but was not statistically significant (*p* = 0.06). Dehydration rates dropped in both groups. RR of being dehydrated in an intervention home vs. control home was 0.36 (*p* = 0.025) Patient Satisfaction: Resident perception of drink enjoyment was slightly higher in control group (*p* = 0.237)
Lin 2013 [31]	Plasma osmolality (mOsm/kg); Lab values; BIA (Ohms); HLEs: not reported Fluid intake (mL): *Intervention:* baseline: 1449 ± 421 mL, post: 1732 ± 301 mL per day. Control: baseline: 1539 ± 565 mL, post: 1548 ± 558 mL per day. Fluid intake was statistically significant in the intervention group (*p* < 0.01). Urinary Indices: Baseline: Intervention: USG 1.012 Control: USG 1.009. Values remained the same post intervention. No *p* value reported.
McCormick et al. 2006 [30]	Plasma osmolality (mOsm/kg); Lab values; BIA (Ohms); Urinary indices: not reported Fluid intake (mL): ‘Usual thickener’: 785 mL per day; Pre-thickened: 795 mL, *p* ≤ 0.47 HLEs: No difference in constipation rates. No *p*-value reported.
Mentes and Culp 2003 [26]	Plasma osmolality (mOsm/kg); Lab values; BIA (Ohms): not reported % meeting fluid goal per day: Intervention: baseline: 99, mean over intervention period: 95 (*p* = 0.08); *Control:* baseline: 107, mean over intervention period: 89 (*p* = 0.08) No amount (mls) reported. Urinary Indices: Intervention and control respectively Baseline: USG (1.0166, 1.0189) (*p* = 0.002), urine colour (2.2, 2.6) Mean over intervention period: USG (1.0163, 1.0178) (*p* = 0.07) urine colour (2.2, 2.8) (*p* = 0.08) HLEs: Intervention: 3 events per 63 days of follow-up; Control: 6 events per 60 days of follow-up. RR = 0.48, 95% CI 0.18–1.26 (*p* = 0.039)
Murray et al. 2016 [24]	Plasma osmolality (mOsm/kg); BIA (Ohms); Urinary indices:not reported Lab values: Baseline: 71% BUN:Cr > 20 (dehydrated) Intervention: trend of improvement (day 0 = 22.46 ± 3.70, day 7 = 21.09 ± 2.47, day 14 = 20.56 ± 3.70). Control: trend of deterioration (day 0 = 20.28 ± 3.88, day 7 = 21.63 ± 7.54, day 14 = 24.70 ± 12.71) (*p* = 0.427) Fluid intake (mL): Intervention: 1103 ± 215 mL (299 mL water); Control: 1103 ± 247 mL, (*p* = 0.998) HLEs: Thickened liquids only group had a significantly higher proportion of UTIs compared to water protocol group (*p* = 0.024) Patient satisfaction: Difference in satisfaction ratings between water and thickened fluids were not significant (*p* = 0.655)
Robinson and Rosher 2002 [22]	Plasma osmolality (mOsm/kg); Lab values; Urinary indices: not reported BIA (Ohms): Fluid in each body compartment increased during intervention and declined after program cessation (*p* = 0.001) Fluid intake (mL): 53% met 450 mL daily goal; 24% did not meet the goal every time. No *p*-value or mL amount reported. HLEs: Increase in number of bowel movements (*p* = 0.04), decline in number of falls (*p* = 0.05) Satisfaction: Positive comments were generally made about the program. No p value was reported.
Schnelle et al. 2010 [33]	Plasma osmolality (mOsm/kg); Lab values; BIA (Ohms); Urinary indices: not reported Fluid intake (mL): Intervention: 399 ± 186 mL; Control: 56.2 ± 118 mL. Significant increase from baseline (baseline values not reported, *p* < 0.001) HLEs: Fewer intervention subjects met the criterion for constipation compared to baseline *p* < 0.001
Simmons, Alessi and Schnelle 2001 [25]	BIA (Ohms); Urinary indices; Number of HLEs: not reported Plasma Osmolality (mOsm/kg): Intervention and control respectively Baseline: 303.6 ± 9.1, 303.4 ± 8.5 8 weeks: 300.5 ± 9.1, 298.6 ± 10.5 32 weeks: 297.0 ± 10.8*, 294.7 ± 11.9 Significant decline in both groups overtime (*p* < 0.05). Lab Values: BUN:Cr ratio (intervention and control respectively) Baseline: 24.0 (±4.6), 21.7 (±6.1); *8 weeks:* 26.2 (±8.8), 22.3 (±5.7); *32 weeks:* 22.9 (±5.6), 23.8 (±7.2) Changes not significant (*p* > 0.05) Fluid Intake (ml): Between meals; Phase 1: 290 ± 136 mL; Phase 2: 476 ± 296 mL; Phase 3: 633 ± 376 mL. Significant increase between phase 1 and 2 (*p* < 0.001). Significant increase between phase 2 and 3 (*p* < 0.001).
Smith et al. 2019 [27]	Plasma osmolality (mOsm/kg); Lab values; BIA (Ohms); Urinary indices; Number of HLEs: not reported Fluid intake (mL): Adjusted mean fluid intake at baseline: 1550.51 mL. Adjusted mean fluid intake post: 2224.81 mL. No *p* value reported.
Spangler et al. 1984 [38]	Plasma osmolality (mOsm/kg); Lab values; BIA (Ohms); Number of HLEs: not reported Urinary Indices: Baseline: 25% of residents had scores >20 (dehydration); Post: All residents had scores <20 (absence of dehydration) *p* < 0.002.
Tanaka et al. 2009 [36]	Plasma osmolality (mOsm/kg); Lab values; BIA (Ohms); Urinary indices; Number of HLEs: not reported Fluid intake (mL): Baseline: 881.1 ± 263.8 per day; Post: 1146.4 ± 365.2 per day, *p* < 0.001.
Taylor and Barr 2006 [37]	Plasma osmolality (mOsm/kg); Lab values; BIA (Ohms); Urinary indices; Number of HLEs: not reported Fluid intake (mL): 3 meal menu: 612 ±176 mL; 5 meal menu: 698 ± 156 mL. Fluid intake was higher with 5 meals vs. 3 meals (*p* = 0.003).
Wilson et al. 2019 [28]	Plasma osmolality (mOsm/kg); Lab values; BIA (Ohms); Urinary indices: not reported Fluid intake (mL): Home A: daily mean fluid intakes <1500 mL; Home B: daily mean fluid intakes >1500 mL. No *p* value or soecific amount (mL) reported. HLEs: No change in HLEs. Significant decrease in average daily use of laxatives at both homes (*p* < 0.05). No change in use of antibiotics.

**Table 4:** Characterisation of interventions using categories from the Behaviour Change Wheel [51].

Intervention Functions	Included Articles
Education	None reported
Persuasion	Lin, Simmons et al., Smith et al., Tanaka et al.
Incentivisation	None reported
Coercion	None reported
Training	None reported
Enablement	Allen et al., Bak et al., Robinson and Rosher, Smith et al., Wilson et al., Mentes and Culp, Howard et al., Karagiannis, Chivers and Karagiannis, Murray et al., McCormick et al., Schnelle et al., Spangler et al.
Modelling	None reported
Environmental restructuring	Dunne et al., Holzapfel et al., Kenkmann et al., Taylor and Barr, Wilson et al.
Restrictions	None reported

## Data Availability

The data presented in this study are available on request from the corresponding author.

## Supplementary Materials

The following are available online at www.mdpi.com/article/10.3390/nu13103640/s1, Table S1: Search terms (CINAHL); Table S2: Risk of Bias Assessment on Included Studies (*n* = 19).
