# Supplementary material for: Interventions to Improve Hydration in Older Adults: A Systematic Review and Meta-Analysis
Source: Nutrients. 2021 Oct 18;13(10):3640. doi: 10.3390/nu13103640 (PMC8537864; doi:10.3390/nu13103640)
Supplement: Supplementary file 1 [file nutrients-13-03640-s001.zip › nutrients-1416142-supplementary.pdf]

Supplementary Table S1. Search terms (CINAHL)

Population search terms	(MH "Aged") OR (MH "Aged, 80 and over") OR (MH "Geriatrics") OR (old or older) N5 (resident* or age* or adult* or person* or people or wom?n or female* or m?n or male* or patient*) OR elder*OR geriatr* OR "nursing home patients" OR "aged hospitalized" OR alzheim* OR dement* OR(MH "Frail Elderly") OR cognitive n3 (impair* OR dysfunction*)
Intervention search terms	(MH "Fluid Therapy") OR (MH "Beverages") ORdrink* OR fluid* N3 therap* OR ((fluid* or water*or liquid*) N5 (intake* or monitor* or replac* or replenish*))
Outcome search terms	(MH "Dehydration") OR (MH "Hypernatremia")OR dehydrat* OR dehydrat* OR hydrat* OR rehydrat* OR hypovolemia OR (MH "Hypovolemia")

Supplementary Table S2: Risk of Bias Assessment of Included Studies (n=19)

Author	Research question	Selection criteria	Comparable groups	Follow-up	Blinding	Intervention	Outcomes	Statistics	Conclusion	Funding	Overall rating
Allen et al. 2013	Y	Y	Y	Y	Y	Y	Y	Y	Y	Y	Positive
Bak et al. 2018	Y	UC	UC	UC	N	UC	UC	N	Y	Y	Neutral
Dunne et al. 2004	Y	UC	NA	UC	N	N	UC	UC	N	Y	Neutral
Holzapfel et al. 1996	Y	Y	UC	UC	N	Y	UC	UC	Y	Y	Neutral
Howard et al. 2018	Y	Y	Y	NA	NA	Y	Y	UC	Y	UC	Positive
Karagiannis et al. 2011	Y	Y	Y	UC	Y	Y	Y	N	Y	Y	Positive
Kenkmann et al. 2010	Y	UC	UC	Y	UC	UC	N	Y	Y	N	Neutral
Lin 2013	Y	UC	Y	Y	UC	Y	Y	UC	Y	Y	Positive
McCormick et al. 2006	Y	UC	Y	Y	UC	UC	UC	N	N	N	Neutral
Mentes & Culp 2003	Y	Y	Y	N	Y	UC	Y	N	Y	Y	Positive
Murray et al. 2016	Y	Y	Y	Y	Y	Y	Y	Y	Y	Y	Positive
Robinson & Rosher 2002	Y	UC	NA	UC	UC	UC	UC	N	N	UC	Neutral
Schanelle et al. 2010	Y	Y	Y	Y	Y	UC	Y	Y	Y	Y	Positive
Simmons et al. 2001	Y	Y	UC	N	Y	Y	Y	Y	Y	UC	Positive

Legend: Y = yes, N = no, UC = unclear, NA = not applicable
